# New Species of *Talaromyces* (Fungi) Isolated from Soil in Southwestern China

**DOI:** 10.3390/biology10080745

**Published:** 2021-08-04

**Authors:** Zhi-Kang Zhang, Xin-Cun Wang, Wen-Ying Zhuang, Xian-Hao Cheng, Peng Zhao

**Affiliations:** 1 School of Agriculture, Ludong University, Yantai 264025, China; zhangzk@m.ldu.edu.cn (Z.-K.Z.); chengxianhao@ldu.edu.cn (X.-H.C.); 2 State Key Laboratory of Mycology, Institute of Microbiology, Chinese Academy of Sciences, Beijing 100101, China; zhuangwy@im.ac.cn

**Keywords:** Ascomycota, biodiversity hotspot, DNA barcodes, Eurotiales, phylogeny, taxonomy, Trichocomaceae

## Abstract

**Simple Summary:**

*Talaromyces* species are distributed all around the world and occur in various environments, e.g., soil, air, living or rotten plants, and indoors. Some of them produce enzymes and pigments of industrial importance, while some cause Talaromycosis. *Talaromyces marneffei*, a well-known and important human pathogen, is endemic to Southeast Asia and causes high mortality, especially in HIV/AIDS patients and those with other immunodeficiencies. China covers 3 of the 35 global biodiversity hotspots. During the explorations of fungal diversity in soil samples collected at different sites of southwestern China, two new *Talaromyces* species, *T. chongqingensis* X.C. Wang and W.Y. Zhuang and *T. wushanicus* X.C. Wang and W.Y. Zhuang, were discovered based on phylogenetic analyses and morphological comparisons. They are described and illustrated in detail. Six phylogenetic trees of the sections *Talaromyces* and *Trachyspermi* were constructed based on three-gene datasets and revealed the phylogenetic positions of the new species. This work provided a better understanding of biodiversity and phylogeny of the genus. The results make the concepts of the two sections of *Talaromyces* well-established. The discovery will be beneficial for future evaluation of the potential usages and functions of the new species.

**Abstract:**

Southwestern China belongs among the global biodiversity hotspots and the Daba Mountains are recognized as one of the priority conservation areas. During the exploration of fungal biodiversity from soil samples collected from Mount Daba, two species of *Talaromyces* were discovered as new to science based on phylogenetic analyses and morphological comparisons. *Talaromyces chongqingensis* sp. nov. is a sister taxon of *T. minioluteus* and *T. minnesotensis* in the section *Trachyspermi*; and *T. wushanicus* sp. nov., affiliated to the section *Talaromyces*, is closely related to *T. cnidii* and *T. siamensis*. The new species differ from their sisters in DNA sequences, growth rates, and morphological characteristics. Descriptions and illustrations of them are provided in detail.

## 1. Introduction

*Talaromyces* C.R. Benj. is a cosmopolitan genus occurring in various environments, e.g., soil, air, living or rotten plants, and indoors. Its beneficial and harmful effects on humans have been well documented. Enzymes and pigments produced by some species of the genus are of industrial importance, such as β-glucosidase produced by *T. amestolkiae* N. Yilmaz et al. [1] and *T. cellulolyticus* T. Fujii et al. [2], and red pigments by *T. atroroseus* N. Yilmaz et al. [3,4]. Talaromycosis caused by several species were also reported [5,6]. Among them, *T. marneffei* (Segretain et al.) Samson et al., endemic to Southeast Asia, is a well-known and important human pathogen causing high mortality in the absence of proper diagnosis and prompt treatment, especially in HIV/AIDS patients and those with other immunodeficiencies [7].

A total of 170 *Talaromyces* species were accepted and classified into seven sections according to a recent monographic study [8]. Moreover, *T. albisclerotius* B.D. Sun et al., *T. aspriconidius* B.D. Sun et al., *T. aureolinus* L. Wang, *T. bannicus* L. Wang, *T. brevis* B.D. Sun et al., *T. guizhouensis* B.D. Sun et al., *T. penicillioides* L. Wang, *T. pulveris* Crous, *T. rufus* B.D. Sun et al., *T. sparsus* L. Wang, *T. tenuis* B.D. Sun et al., and *T. yunnanensis* Doilom and C.F. Liao were later described [9,10,11,12]. In the section (sect.) *Trachyspermi* Yaguchi and Udagawa, 30 species are commonly accepted; and in the sect. *Talaromyces*, the largest part of the genus, 75 species have been recognized.

During the explorations of fungal diversity in soil samples collected at different sites of Chongqing and Sichuan in southwestern China, two *Talaromyces* species belonging to the sections *Talaromyces* and *Trachyspermi* were further discovered as new to science based on phylogenetic analyses and morphological comparisons. They are described and illustrated in detail.

## 2. Materials and Methods

### 2.1. Fungal Materials

Cultures were isolated from soil samples collected from Chongqing and areas nearby in Sichuan Province in October 2020. Dried cultures were deposited in the Herbarium Mycologicum Academiae Sinicae (HMAS), and the living ex-type strains were preserved in the China General Microbiological Culture Collection Center (CGMCC).

### 2.2. Morphological Observations

Morphological characterization was conducted following the standardized methods [13]. Four standard growth media were used: Czapek yeast autolysate agar (CYA, yeast extract Oxoid, Hampshire, UK), malt extract agar (MEA, Amresco, Solon, OH, USA), yeast extract agar (YES) and potato dextrose agar (PDA). The methods for inoculation, incubation, microscopic examinations, and digital recordings were following our previous studies [14,15,16].

### 2.3. DNA Extraction, PCR Amplification, and Sequencing

DNA was extracted from the cultures grown on PDA for 7 days using the Plant Genomic DNA Kit (DP305, TIANGEN Biotech, Beijing, China). Polymerase chain reaction (PCR) amplifications of the internal transcribed spacer (ITS), beta-tubulin (BenA), calmodulin (CaM) and RNA polymerase II second largest subunit (RPB2) gene regions were conducted with routine methods [14,15,16]. The products were purified and subject to sequencing on an ABI 3730 DNA Sequencer (Applied Biosystems, Bedford, MA, USA). Although the ITS region, the recommended standard DNA barcode for fungi, is not sufficient to discriminate the species of this genus, the sequences provided here will be helpful for other researchers in case of need.

### 2.4. Phylogenetic Analyses

The forward and reverse sequences newly generated in this study were assembled using Seqman v. 7.1.0 (DNASTAR Inc., Madison, WI, USA). The assembled sequences were deposited in GenBank. Previously described species from the corresponding sections, which were used for phylogenetic analyses, are listed in Table 1 and Table 2. Newly generated sequences of this study are shown in Table 3. For each section, three datasets of BenA, CaM, and RPB2 were compiled. Sequences of each dataset (35 species for sect. *Trachyspermi* and 79 species for sect. *Talaromyces*) were aligned using MAFFT v. 7.221 [17], and then manually edited in BioEdit v. 7.1.10 [18] and MEGA v. 6.0.6 [19]. Maximum likelihood (ML) analyses were performed using RAxML-HPC2 [20] on XSEDE 8.2.12 on CIPRES Science Gateway v. 3.3 [21] with the default GTRCAT model. Bayesian Inference (BI) analyses were performed with MrBayes v. 3.2.5 [22]. Appropriate nucleotide substitution models and parameters were determined by Modeltest v. 3.7 [23]. The consensus trees were viewed in FigTree v. 1.3.1 (Available online: http://tree.bio.ed.ac.uk/software/figtree/ (accessed on 1 September 2015)). The type species of section *Talaromyces* served as outgroup taxon of the *Trachyspermi* tree and vice versa.

## 3. Results

### 3.1. Phylogenetic Analysis

**Figure 1:**
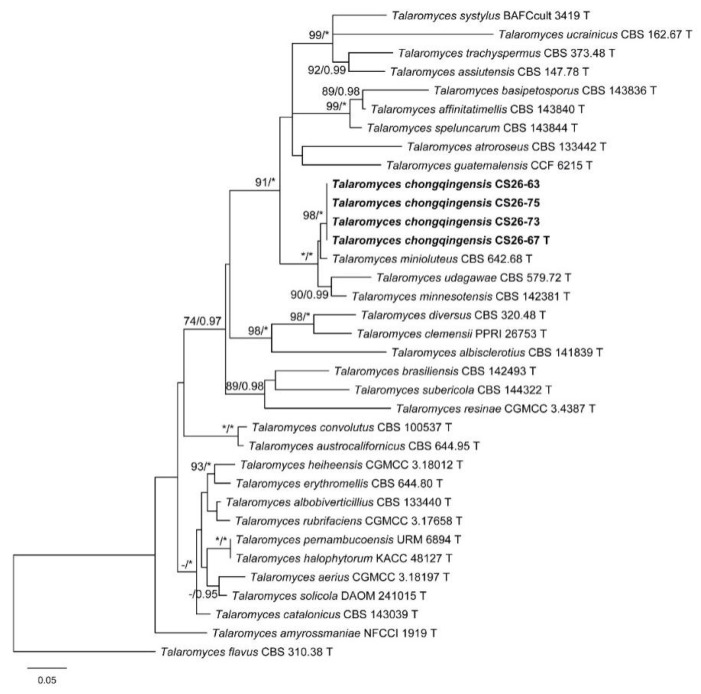
Maximum likelihood phylogeny of *Talaromyces* sect. *Trachyspermi* inferred from the BenA dataset. Bootstrap values ≥ 70% (**left**) or posterior probability values ≥ 0.95 (**right**) are indicated at nodes. Asterisk denotes 100% bootstrap or 1.00 posterior probability.

**Figure 2:**
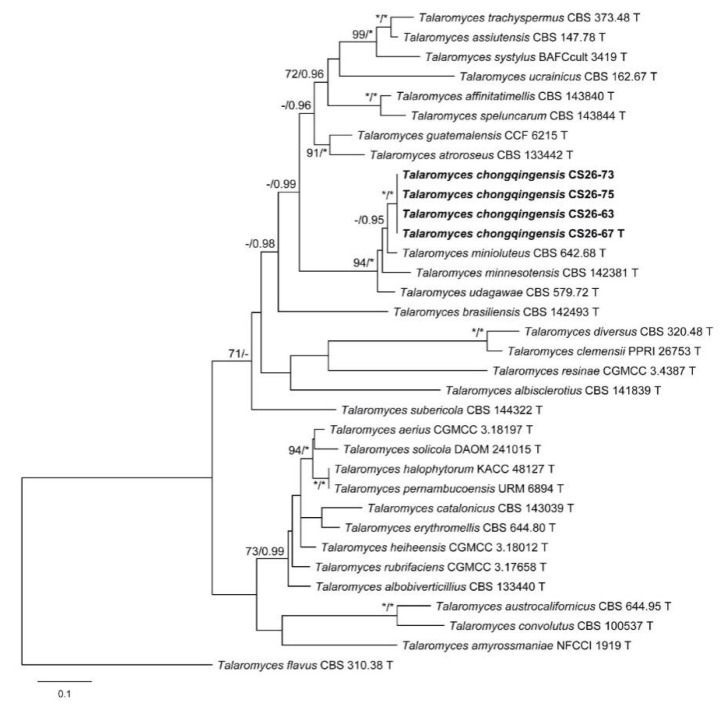
Maximum likelihood phylogeny of *Talaromyces* sect. *Trachyspermi* inferred from the CaM dataset. Bootstrap values ≥ 70% (**left**) or posterior probability values ≥ 0.95 (**right**) are indicated at nodes. Asterisk denotes 100% bootstrap or 1.00 posterior probability.

**Figure 3:**
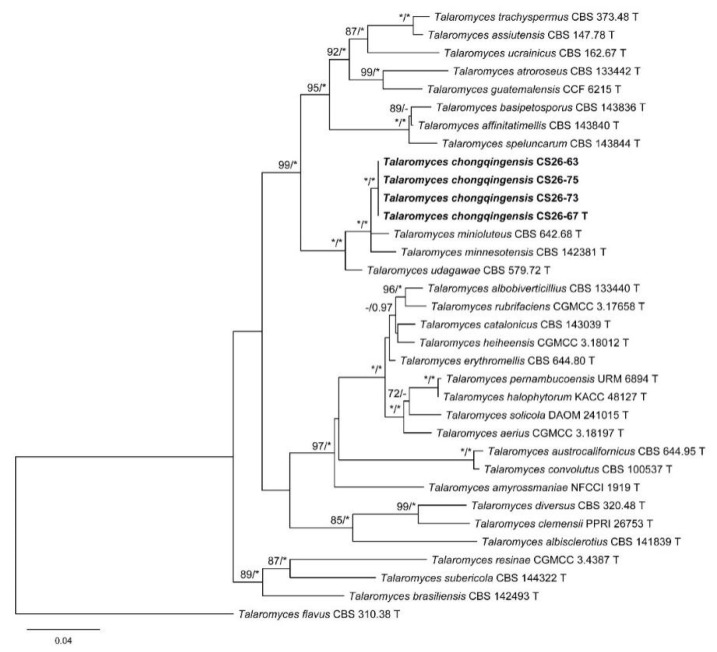
Maximum likelihood phylogeny of *Talaromyces* sect. *Trachyspermi* inferred from the RPB2 dataset. Bootstrap values ≥ 70% (**left**) or posterior probability values ≥ 0.95 (**right**) are indicated at nodes. Asterisk denotes 100% bootstrap or 1.00 posterior probability.

**Figure 4:**
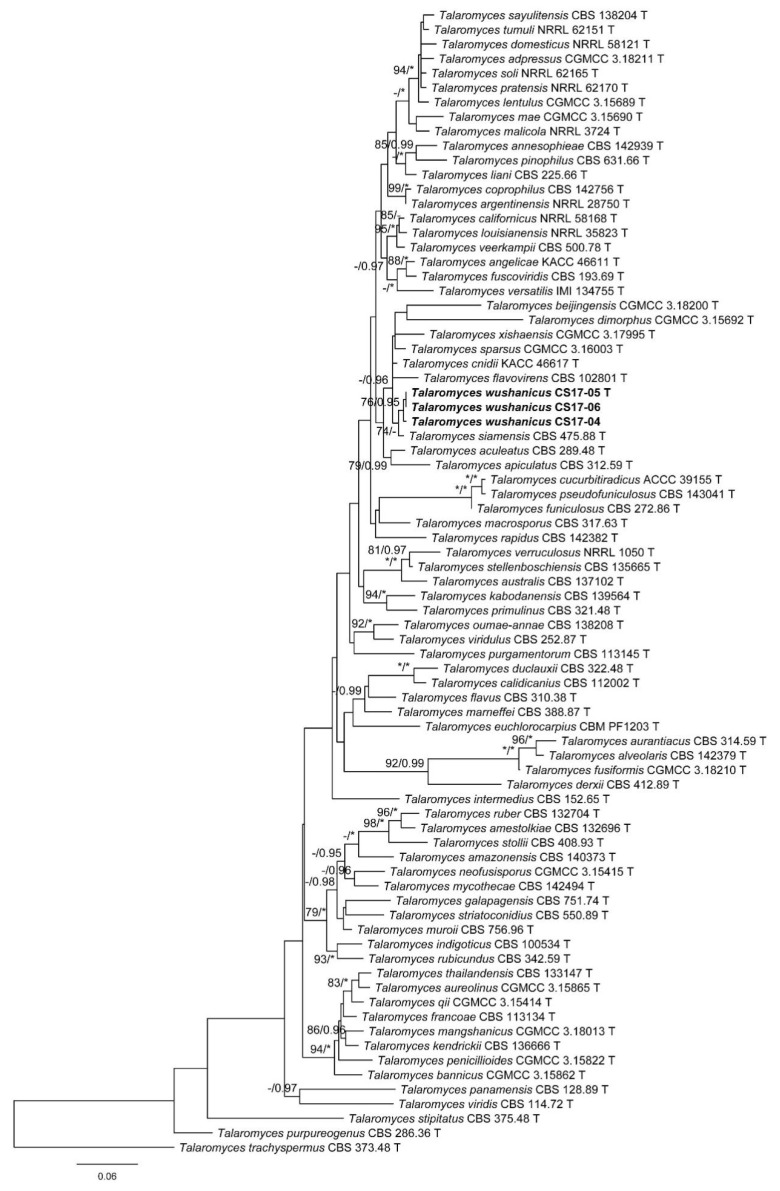
Maximum likelihood phylogeny of *Talaromyces* sect. *Talaromyces* inferred from the BenA dataset. Bootstrap values ≥ 70% (**left**) or posterior probability values ≥ 0.95 (**right**) are indicated at nodes. Asterisk denotes 100% bootstrap or 1.00 posterior probability.

**Figure 5:**
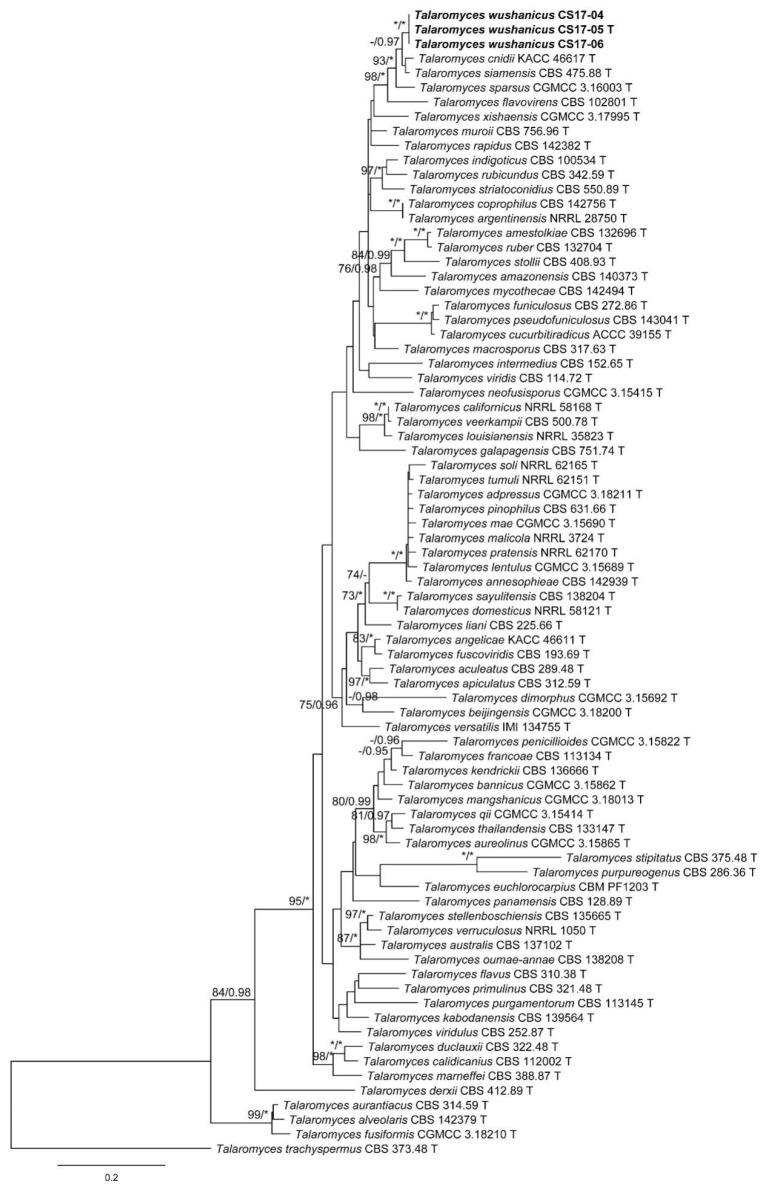
Maximum likelihood phylogeny of *Talaromyces* sect. *Talaromyces* inferred from the CaM dataset. Bootstrap values ≥ 70% (**left**) or posterior probability values ≥ 0.95 (**right**) are indicated at nodes. Asterisk denotes 100% bootstrap or 1.00 posterior probability.

**Figure 6:**
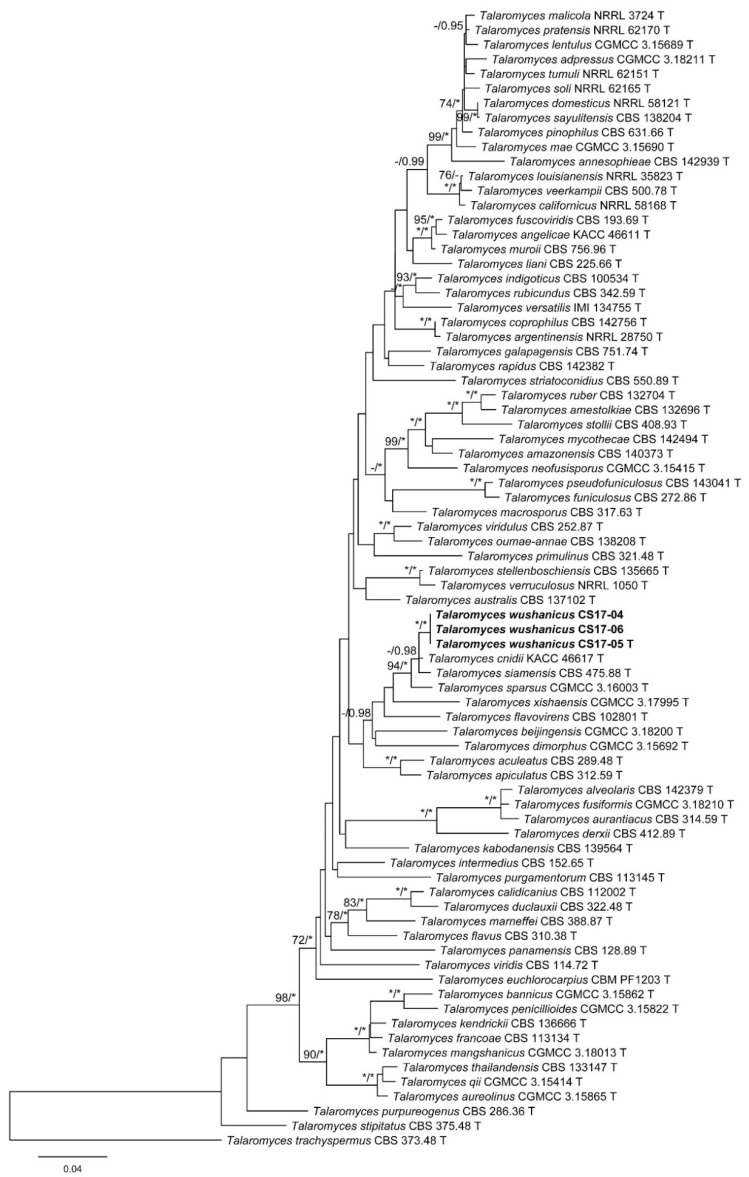
Maximum likelihood phylogeny of *Talaromyces* sect. *Talaromyces* inferred from the RPB2 dataset. Bootstrap values ≥ 70% (**left**) or posterior probability values ≥ 0.95 (**right**) are indicated at nodes. Asterisk denotes 100% bootstrap or 1.00 posterior probability.

The characteristics of datasets used in the phylogenetic analyses are presented in Table 4. Phylogenetic analyses of the section *Trachyspermi* revealed that *T. chongqingensis* always grouped with *T. minioluteus*, *T. minnesotensis*, and *T. udagawae*, having strong statistic supports. In the BenA and CaM analyses (Figure 1 and Figure 2), *T. minioluteus* was the closest sister of the new species; while *T. minioluteus* and *T. minnesotensis* were both closely related to *T. chongqingensis* in the RPB2 tree (Figure 3). In the phylogenetic analysis of section *Talaromyces* based on the BenA dataset, *T. wushanicus* clustered with *T. siamensis* (Figure 4); while *T. cnidii* and *T. siamensis* were closely related to the new species in the CaM and RPB2 analyses (Figure 5 and Figure 6).

### 3.2. Taxonomy

**Figure 7:**
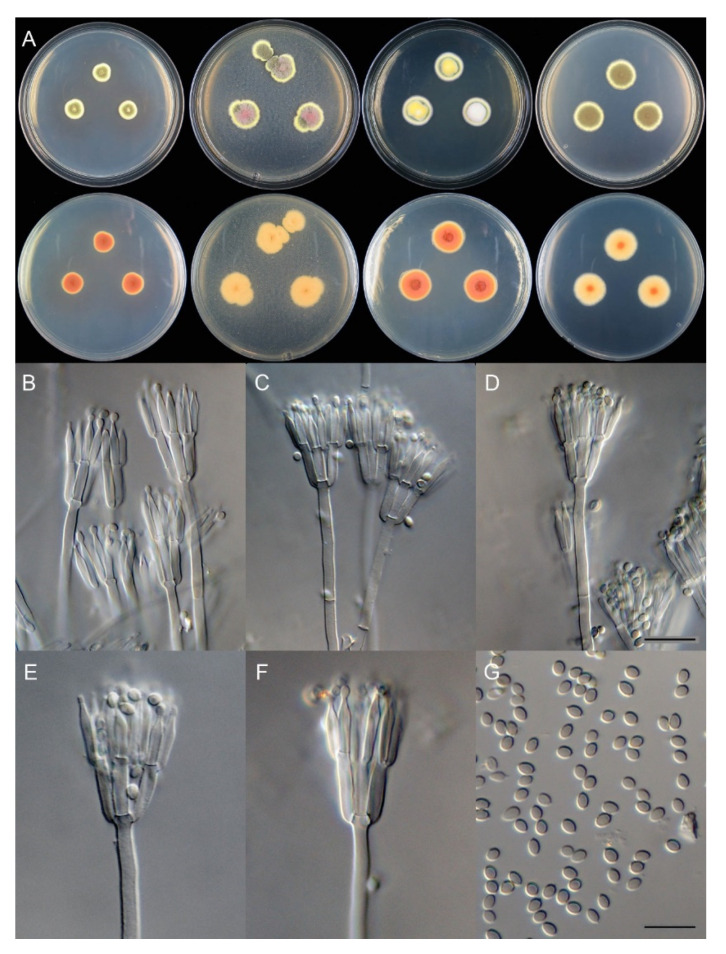
*Talaromyces chongqingensis* (CS26-67). (**A**) Colonies: top row left to right, obverse CYA, MEA, YES, and PDA; bottom row left to right, reverse CYA, MEA, YES, and PDA; (**B**–**F**) Conidiophores; (**G**) Conidia. Bars: (**D**) 15 µm, applies also to (**B**,**C**); (**G**) 10 µm, applies also to (**E**,**F**).

*Talaromyces chongqingensis* X.C. Wang and W.Y. Zhuang, sp. nov., Figure 7.

Fungal Names: FN570851

Etymology: The specific epithet refers to the type locality.

in Talaromyces sect. Trachyspermi

Typification: China, Chongqing City, Chengkou County, Daba Mountain National Nature Reserve, Gaoguan Town, at the riverside of River Ren, 31°49′40′′ N 109°0′24′′ E, in soil under a palm tree, 30 October 2020, Xin-Cun Wang, Huan-Di Zheng and Chang Liu, culture, Zhi-Kang Zhang, CS26-67 (holotype HMAS 247849, ex-type strain CGMCC 3.20482).

DNA barcodes: ITS MZ358001, BenA MZ361343, CaM MZ361350, RPB2 MZ361357.

Colony diam: after 7 days at 25 °C (unless stated otherwise): on CYA, 12–13 mm; on CYA at 37 °C, no growth; on CYA at 5 °C, no growth; on MEA, 17–18 mm; on YES 18–19 mm; on PDA, 18–19 mm.

Colony characteristics: 

On CYA at 25 °C, after 7 days: colonies nearly circular, protuberant in centers; margins moderately wide, entire; mycelia white and yellow; texture velutinous; sporulation dense; conidia *en masse* yellowish green to dull green; soluble pigments light brown; exudates small, clear; reverse orange, buff at the margins but dark orange at centers.

On MEA at 25 °C, after 7 days: Colonies irregular, protuberant in centers, pink hyphae growing at centers; margins moderately wide, irregular; mycelia white and yellow; texture floccose; sporulation dense; conidia *en masse* greyish green; soluble pigments absent; exudates absent; reverse buff.

On YES at 25 °C, after 7 days: Colonies nearly circular, strongly protuberant in centers; margins moderately wide, entire; mycelia white and yellow; texture velutinous; sporulation moderately dense; conidia *en masse* pale green; soluble pigments light brown; exudates absent; reverse orange, yellow brown at the margins but dark orange at centers.

On PDA at 25 °C, after 7 days: Colonies nearly circular, plain, slightly protuberant in centers; margins moderately wide, irregular; mycelia white and yellow; texture velutinous; sporulation very dense; conidia *en masse* yellowish green; soluble pigments absent; exudates absent; reverse yellow brown, buff at the margins but orange at centers.

Micromorphology: Conidiophores biverticillate; stipes smooth-walled, 90–250 × 2.5–3.0 μm; metulae 4–5, 10–13 × 2.5–3.5 μm; phialides acerose, tapering into very thin neck, 3–5 per metula, 10–13.5 × 2.0–2.5 μm; conidia ellipsoidal to broad fusiform, smooth-walled, 2.5–3.5 × 2.0–2.5 μm.

Additional strains examined: China, Chongqing City, Chengkou County, Daba Mountain National Nature Reserve, Gaoguan Town, at the riverside of River Ren, 31°49′40″ N 109°0′24″ E, in soil under a palm tree, 30 October 2020, Xin-Cun Wang, Huan-Di Zheng and Chang Liu, culture, Zhi-Kang Zhang, CS26-63; *ibid.*, CS26-73; *ibid.*, CS26-75.

Notes: This species is phylogenetically close to *T. minioluteus* and *T. minnesotensis*, but differs from them in growth rate on CYA and MEA at 25 °C (Table 5) and pink hyphae present at the centers of colonies on MEA. The sequence data of the four cultures of this fungus are completely identical.

**Figure 8:**
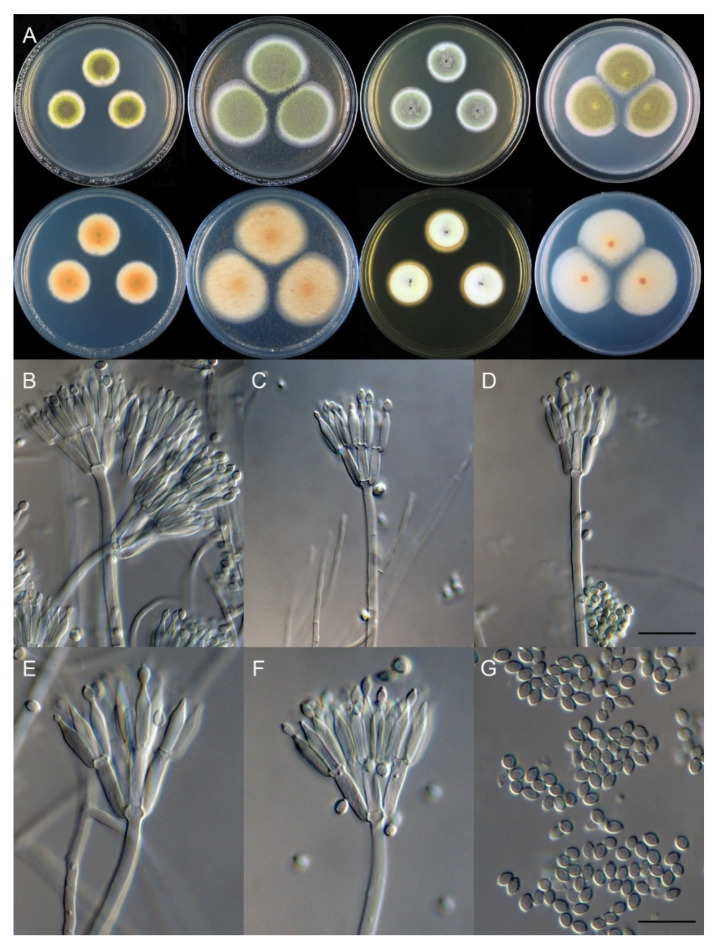
*Talaromyces wushanicus* (CS17-05). (**A**) Colonies: top row left to right, obverse CYA, MEA, YES, and PDA; bottom row left to right, reverse CYA, MEA, YES, and PDA; (**B**–**F**) Conidiophores; (**G**) Conidia. Bars: (**D**) 15 µm, applies also to (**B**,**C**); (**G**) 10 µm, applies also to (**E**,**F**).

*Talaromyces wushanicus* X.C. Wang and W.Y. Zhuang, sp. nov., Figure 8.

Fungal Names: FN570852

Etymology: The specific epithet refers to the type locality.

in Talaromyces sect. Talaromyces

Typification: China, Chongqing City, Wushan County, Dachang Town, Yanghe Village, 31°17′33′′ N 109°50′44′′ E, in soil, 29 October 2020, Xin-Cun Wang, Huan-Di Zheng and Chang Liu, culture, Zhi-Kang Zhang, CS17-05 (holotype HMAS 247848, ex-type strain CGMCC 3.20481).

DNA barcodes: ITS MZ356356, BenA MZ361347, CaM MZ361354, RPB2 MZ361361.

Colony diam: after 7 days at 25 °C (unless stated otherwise): on CYA, 21–24 mm; on CYA at 37 °C, 17–19 mm; on CYA at 5 °C, no growth; on MEA, 40–44 mm; on YES, 24–28 mm; on PDA, 37–38 mm.

Colony characteristics: On CYA 25 °C, 7 days: Colonies nearly circular, protuberant in centers; margins narrow to moderately wide, nearly entire; mycelia white; texture velutinous; sporulation moderately dense; conidia *en masse* yellowish green; soluble pigments absent; exudates almost absent, sometimes very tiny, red, clear; reverse buff, orange to light brown at centers, but white and pink at periphery.

On CYA at 37 °C, after 7 days: Colonies irregular or nearly circular, protuberant in centers; margins moderately wide, nearly entire; mycelia white; texture velutinous; sporulation moderately dense; conidia *en masse* dull green to greyish green; soluble pigments absent; exudates absent; reverse buff.

On MEA at 25 °C, after 7 days: Colonies nearly circular, plain; margins wide, entire; mycelia yellow; texture velutinous; sporulation dense; conidia *en masse* yellowish green; soluble pigments absent; exudates almost absent, sometimes very tiny, hyaline, clear; reverse buff, but yellow to orange in centers.

On YES at 25 °C, after 7 days: Colonies nearly circular, deep, wrinkled, highly protuberant in centers; margins narrow to moderately wide, entire; mycelia white; texture velutinous; sporulation dense; conidia *en masse* yellowish green to dark green; soluble pigments absent; exudates absent, rarely red and clear; reverse white, yellow brown to light brown, rimose, or deeply concave in centers.

On PDA at 25 °C, after 7 days: Colonies nearly circular, plain, slightly protuberant in centers; margins moderately wide, entire; mycelia white; texture velutinous; sporulation dense; conidia *en masse* yellowish green; soluble pigments absent; exudates hyaline, clear, present at centers; reverse greyish white to greenish white, reddish brown at centers.

Micromorphology: Conidiophores biverticillate, rarely terverticillate; stipes smooth-walled, 85–225 × 2.0–3.0 μm; metulae 5, 9.5–11.5 × 2.5–3.0 μm; phialides acerose, tapering into very thin neck, 3–4 per metula, 10–11 × 2.0–2.5 μm; conidia ellipsoidal to broad fusiform, smooth to finely rough, 3–4 × 2.5–3 μm.

Additional strains examined: China, Chongqing City, Wushan County, Dachang Town, Yanghe Village, 31°17′33′′ N 109°50′44′′ E, in soil, 29 October 2020, Xin-Cun Wang, Huan-Di Zheng and Chang Liu, culture, Zhi-Kang Zhang, CS17-04; *ibid.*, CS17-06.

Notes: This species is closely related to *T. cnidii* and *T. siamensis* in the phylogenetic trees (Figure 4, Figure 5 and Figure 6), but it differs from *T. cnidii* in obviously slower growth rate on CYA and YES at 25 °C and from *T. siamensis* by an obviously faster growth on MEA at 25 °C (Table 5). Sequence comparisons indicate that the isolate CS17-04 has a one-base difference in ITS and a two-base difference in BenA from the other two strains. No morphological diversification was found among the strains.

## 4. Discussion

Of the 35 global biodiversity hotspots, 3 are located in southwestern China, consisting of Chongqing, Guizhou, Sichuan, Tibet, and Yunnan provinces [26]. Eight hotspot regions in the southwest of China were identified as priority conservation areas, including the Daba Mountains [27] where materials used for this study were gathered. Soil samples for floristic studies of fungi were collected from Chengkou, Wushan, and Wuxi counties in Chongqing and Wanyuan City in Sichuan. Although *Talaromyces* is a widespread genus and distributed in more than 27 provinces, cities, or regions of China [14], it has never been reported from the above areas.

In recent years, the number of new species of *Talaromyces* increased dramatically. There were 12 species recorded in *Talaromyces* sect. *Trachyspermi* and 36 ones in *Talaromyces* sect. *Talaromyces* in 2014 [24]. From 2018 to 2021, 13 additional species were discovered in the former section, and 20 new members were described in the latter. We are witnessing a trend: new fungal species are described at an accelerated rate.

*Talaromyces* species occur in diversified environments. When the information about the extype strains of more than 100 species in these two sections is gathered (Table 1 and Table 2), it is found that soil is commonly the substrate. Fifty or so species were isolated from different kinds of soil, e.g., forest, cultivated, and swamp soil. Plant debris appears to be the second frequent source, which nearly 20 species inhabited. Four species were from humans and one, the well-known *T. marneffei*, from bamboo rat. Surprisingly, *T. pinophilus* was discovered on PVC, the third widely used plastic in the world, which is hard to biodegrade.

Among the 30 species accepted in *Talaromyces* sect. *Trachyspermi*, 6 were originally reported from China (Table 1). Moreover, 18 of the 75 species known in *Talaromyces* sect. *Talaromyces* were described based on the Chinese samples or specimens (Table 2). These data surely demonstrate that China has a high fungal diversity. With more investigations conducted, we expect to discover more new species of this group of fungi.

## 5. Conclusions

The present work provides a better understanding of biodiversity and phylogeny of the genus. The results make the concepts of the two sections of *Talaromyces* well-established and more sophisticated. The discovery will be beneficial for future evaluation of the potential usages and functions of the new species.

## Figures and Tables

**Table 1:** Previously described *Talaromyces* species used in phylogenetic analyses of the sect. *Trachyspermi*.

Species	Strain	Locality	Substrate	ITS	BenA	CaM	RPB2
*T. aerius* A.J. Chen et al. 2016	CGMCC 3.18197 T	China: Beijing	indoor air	KU866647	KU866835	KU866731	KU866991
*T. affinitatimellis* Rodr.-Andr. et al. 2019	CBS 143840 T	Spain	honey	LT906543	LT906552	LT906549	LT906546
*T. albisclerotius* B.D. Sun et al. 2020	CBS 141839 T	China: Guizhou	soil	MN864276	MN863345	MN863322	MN863334
*T. albobiverticillius* (H.M. Hsieh et al.) Samson et al. 2011	CBS 133440 T	China: Taiwan	decaying leaves	HQ605705	KF114778	KJ885258	KM023310
*T. amyrossmaniae* Rajeshkumar et al. 2019	NFCCI 1919 T	India	decaying fruits of *Terminalia bellerica*	MH909062	MH909064	MH909068	MH909066
*T. assiutensis* Samson and Abdel-Fattah 1978	CBS 147.78 T	Egypt	soil	JN899323	KJ865720	KJ885260	KM023305
*T. atroroseus* N. Yilmaz et al. 2013	CBS 133442 T	South Africa	house dust	KF114747	KF114789	KJ775418	KM023288
*T. austrocalifornicus* Yaguchi and Udagawa 1993	CBS 644.95 T	USA	soil	JN899357	KJ865732	KJ885261	MN969147
*T. basipetosporus* Stchigel et al. 2019	CBS 143836 T	Argentina	honey	LT906542	LT906563	n.a.	LT906545
*T. brasiliensis* R.N. Barbosa et al. 2018	CBS 142493 T	Brazil	honey	MF278323	LT855560	LT855563	MN969198
*T. catalonicus* Guevara-Suarez et al. 2020	CBS 143039 T	Spain	herbivore dung	LT899793	LT898318	LT899775	LT899811
*T. clemensii* Visagie and N. Yilmaz 2019	PPRI 26753 T	South Africa	wood in mine	MK951940	MK951833	MK951906	MN418451
*T. convolutus* Udagawa 1993	CBS 100537 T	Nepal	soil	JN899330	KF114773	MN969316	JN121414
*T. diversus* (Raper and Fennell) Samson et al. 2011	CBS 320.48 T	USA	mouldy leather	KJ865740	KJ865723	KJ885268	KM023285
*T. erythromellis* (A.D. Hocking) Samson et al. 2011	CBS 644.80 T	Australia	soil	JN899383	HQ156945	KJ885270	KM023290
*T. guatemalensis* A. Nováková et al. 2019	CCF 6215 T	Guatemala	soil	MN322789	MN329687	MN329688	MN329689
*T. halophytorum* Y.H. You and S.B. Hong 2020	KACC 48127 T	South Korea	roots of *Limonium tetragonum*	MH725786	MH729367	MK111426	MK111427
*T. heiheensis* X.C. Wang and W.Y. Zhuang 2017	CGMCC 3.18012 T	China: Heilongjiang	rotten wood	KX447526	KX447525	KX447532	KX447529
*T. minioluteus* (Dierckx) Samson et al. 2011	CBS 642.68 T	unknown	unknown	JN899346	MN969409	KJ885273	JF417443
*T. minnesotensis* Guevara-Suarez et al. 2017	CBS 142381 T	USA	human ear	LT558966	LT559083	LT795604	LT795605
*T. pernambucoensis* R. Cruz et al. 2019	URM 6894 T	Brazil	soil	LR535947	LR535945	LR535946	LR535948
*T. resinae* (Z.T. Qi and H.Z. Kong) Houbraken and X.C. Wang 2020	CGMCC 3.4387 T	China: Guizhou	resin of *Eucalyptus tereticornis*	MT079858	MN969442	MT066184	MN969221
*T. rubrifaciens* W.W. Gao 2016	CGMCC 3.17658 T	China: Beijing	hospital air	KR855658	KR855648	KR855653	KR855663
*T. solicola* Visagie and K. Jacobs 2012	DAOM 241015 T	South Africa	soil	FJ160264	GU385731	KJ885279	KM023295
*T. speluncarum* Rodr.-Andr. et al. 2020	CBS 143844 T	Spain	sparkling wine	LT985890	LT985901	LT985906	LT985911
*T. subericola* Rodr.-Andr. et al. 2020	CBS 144322 T	Spain	sparkling wine	LT985888	LT985899	LT985904	LT985909
*T. systylus* S.M. Romero et al. 2015	BAFCcult 3419 T	Argentina	soil	KP026917	KR233838	KR233837	n.a.
*T. trachyspermus* (Shear) Stolk and Samson 1973	CBS 373.48 T	USA	unknown	JN899354	KF114803	KJ885281	JF417432
*T. ucrainicus* (Panas.) Udagawa 1966	CBS 162.67 T	Ukraine	potato starch	JN899394	KF114771	KJ885282	KM023289
*T. udagawae* Stolk and Samson 1972	CBS 579.72 T	Japan	soil	JN899350	KF114796	KX961260	MN969148
*T. flavus* (Klöcker) Stolk and Samson 1972	CBS 310.38 T	New Zealand	unknown	JN899360	JX494302	KF741949	JF417426

**Table 2:** Previously described *Talaromyces* species used in phylogenetic analyses of the sect. *Talaromyces*.

Species	Strain	Locality	Substrate	ITS	BenA	CaM	RPB2
*T. aculeatus* (Raper and Fennell) Samson et al. 2011	CBS 289.48 T	USA	textile	KF741995	KF741929	KF741975	MH793099
*T. adpressus* A.J. Chen et al. 2016	CGMCC 3.18211 T	China: Beijing	indoor air	KU866657	KU866844	KU866741	KU867001
*T. alveolaris* Guevara-Suarez et al. 2017	CBS 142379 T	USA	human bronchoalveolar lavage	LT558969	LT559086	LT795596	LT795597
*T. amazonensis* N. Yilmaz et al. 2016	CBS 140373 T	Colombia	leaf litter	KX011509	KX011490	KX011502	MN969186
*T. amestolkiae* N. Yilmaz et al. 2012	CBS 132696 T	South Africa	house dust	JX315660	JX315623	KF741937	JX315698
*T. angelicae* S.H. Yu et al. 2013	KACC 46611 T	South Korea	dried root of *Angelica gigas*	KF183638	KF183640	KJ885259	KX961275
*T. annesophieae* Houbraken 2017	CBS 142939 T	Netherlands	soil	MF574592	MF590098	MF590104	MN969199
*T. apiculatus* Samson et al. 2011	CBS 312.59 T	Japan	soil	JN899375	KF741916	KF741950	KM023287
*T. argentinensis* Jurjević and S.W. Peterson 2019	NRRL 28750 T	Ghana	soil	MH793045	MH792917	MH792981	MH793108
*T. aurantiacus* (J.H. Mill. et al.) Samson et al. 2011	CBS 314.59 T	USA	soil	JN899380	KF741917	KF741951	KX961285
*T. aureolinus* L. Wang 2021	CGMCC 3.15865 T	China: Yunnan	soil	MK837953	MK837937	MK837945	MK837961
*T. australis* Visagie et al. 2015	CBS 137102 T	Australia	soil under pasture	KF741991	KF741922	KF741971	KX961284
*T. bannicus* L. Wang 2021	CGMCC 3.15862 T	China: Yunnan	soil	MK837955	MK837939	MK837947	MK837963
*T. beijingensis* A.J. Chen et al. 2016	CGMCC 3.18200 T	China: Beijing	indoor air	KU866649	KU866837	KU866733	KU866993
*T. calidicanius* (J.L. Chen) Samson et al. 2011	CBS 112002 T	China: Taiwan	soil	JN899319	HQ156944	KF741934	KM023311
*T. californicus* Jurjević and S.W. Peterson 2019	NRRL 58168 T	USA	air	MH793056	MH792928	MH792992	MH793119
*T. cnidii* S.H. Yu et al. 2013	KACC 46617 T	South Korea	dried roots of *Cnidium* sp.	KF183639	KF183641	KJ885266	KM023299
*T. coprophilus* Guevara-Suarez et al. 2020	CBS 142756 T	Spain	herbivore dung	LT899794	LT898319	LT899776	LT899812
*T. cucurbitiradicus* L. Su and Y.C. Niu 2018	ACCC 39155 T	China: Beijing	endophyte from root of pumpkin (*Cucurbita moschata*)	KY053254	KY053228	KY053246	n.a.
*T. derxii* Takada and Udagawa 1988	CBS 412.89 T	Japan	cultivated soil	JN899327	JX494306	KF741959	KM023282
*T. dimorphus* X.Z. Jiang and L. Wang 2018	CGMCC 3.15692 T	China: Hainan	forest soil	KY007095	KY007111	KY007103	KY112593
*T. domesticus* Jurjević and S.W. Peterson 2019	NRRL 58121 T	USA	floor swab	MH793055	MH792927	MH792991	MH793118
*T. duclauxii* (Delacr.) Samson et al. 2011	CBS 322.48 T	France	canvas	JN899342	JX091384	KF741955	JN121491
*T. euchlorocarpius* Yaguchi et al. 1999	CBM PF1203 T	Japan	soil	AB176617	KJ865733	KJ885271	KM023303
*T. flavovirens* (Durieu and Mont.) Visagie et al. 2012	CBS 102801 T	Spain	unknown	JN899392	JX091376	KF741933	KX961283
*T. flavus* (Klöcker) Stolk and Samson 1972	CBS 310.38 T	New Zealand	unknown	JN899360	JX494302	KF741949	JF417426
*T. francoae* N. Yilmaz et al. 2016	CBS 113134 T	Colombia	leaf litter	KX011510	KX011489	KX011501	MN969188
*T. funiculosus* (Thom) Samson et al. 2011	CBS 272.86 T	India	*Lagenaria vulgaris*	JN899377	MN969408	KF741945	KM023293
*T. fuscoviridis* Visagie et al. 2015	CBS 193.69 T	Netherlands	soil	KF741979	KF741912	KF741942	MN969156
*T. fusiformis* A.J. Chen et al. 2016	CGMCC 3.18210 T	China: Beijing	indoor air	KU866656	KU866843	KU866740	KU867000
*T. galapagensis* Samson and Mahoney 1977	CBS 751.74 T	Ecuador	soil under *Maytenus obovata*	JN899358	JX091388	KF741966	KX961280
*T. indigoticus* Takada and Udagawa 1993	CBS 100534 T	Japan	soil	JN899331	JX494308	KF741931	KX961278
*T. intermedius* (Apinis) Stolk and Samson 1972	CBS 152.65 T	UK	swamp soil	JN899332	JX091387	KJ885290	KX961282
*T. kabodanensis* Houbraken et al. 2016	CBS 139564 T	Iran	hypersaline soil	KP851981	KP851986	KP851995	MN969190
*T. kendrickii* Visagie et al. 2015	CBS 136666 T	Canada	forest soil	KF741987	KF741921	KF741967	MN969158
*T. lentulus* X.Z. Jiang and L. Wang 2018	CGMCC 3.15689 T	China: Shandong	soil	KY007088	KY007104	KY007096	KY112586
*T. liani* (Kamyschko) N. Yilmaz et al. 2014	CBS 225.66 T	China	soil	JN899395	JX091380	KJ885257	KX961277
*T. louisianensis* Jurjević and S.W. Peterson 2019	NRRL 35823 T	USA	air	MH793052	MH792924	MH792988	MH793115
*T. macrosporus* (Stolk and Samson) Frisvad et al. 1990	CBS 317.63 T	South Africa	apple juice	JN899333	JX091382	KF741952	KM023292
*T. mae* X.Z. Jiang and L. Wang 2018	CGMCC 3.15690 T	China: Shanghai	forest soil	KY007090	KY007106	KY007098	KY112588
*T. malicola* Jurjević and S.W. Peterson 2019	NRRL 3724 T	Italy	rhizosphere of an apple tree	MH909513	MH909406	MH909459	MH909567
*T. mangshanicus* X.C. Wang and W.Y. Zhuang 2016	CGMCC 3.18013 T	China: Hunan	soil	KX447531	KX447530	KX447528	KX447527
*T. marneffei* (Segretain et al.) Samson et al. 2011	CBS 388.87 T	Vietnam	bamboo rat (*Rhizomys sinensis*)	JN899344	JX091389	KF741958	KM023283
*T. muroii* Yaguchi et al. 1994	CBS 756.96 T	China: Taiwan	soil	MN431394	KJ865727	KJ885274	KX961276
*T. mycothecae* R.N. Barbosa et al. 2018	CBS 142494 T	Brazil	nest of stingless bee (*Melipona scutellaris*)	MF278326	LT855561	LT855564	LT855567
*T. neofusisporus* L. Wang 2016	CGMCC 3.15415 T	China: Tibet	leaf sample	KP765385	KP765381	KP765383	MN969165
*T. oumae-annae* Visagie et al. 2014	CBS 138208 T	South Africa	house dust	KJ775720	KJ775213	KJ775425	KX961281
*T. panamensis* (Samson et al.) Samson et al. 2011	CBS 128.89 T	Panama	soil	JN899362	HQ156948	KF741936	KM023284
*T. penicillioides* L. Wang 2021	CGMCC 3.15822 T	China: Guizhou	soil	MK837956	MK837940	MK837948	MK837964
*T. pinophilus* (Hedgc.) Samson et al. 2011	CBS 631.66 T	France	PVC	JN899382	JX091381	KF741964	KM023291
*T. pratensis* Jurjević and S.W. Peterson 2019	NRRL 62170 T	USA	effluent of water treatment plant	MH793075	MH792948	MH793012	MH793139
*T. primulinus* (Pitt) Samson et al. 2011	CBS 321.48 T	USA	unknown	JN899317	JX494305	KF741954	KM023294
*T. pseudofuniculosus* Guevara-Suarez et al. 2020	CBS 143041 T	Spain	herbivore dung	LT899796	LT898323	LT899778	LT899814
*T. purgamentorum* N. Yilmaz et al. 2016	CBS 113145 T	Colombia	leaf litter	KX011504	KX011487	KX011500	MN969189
*T. purpureogenus* (Stoll) Samson et al. 2011	CBS 286.36 T	unknown	unknown	JN899372	JX315639	KF741947	JX315709
*T. qii* L. Wang 2016	CGMCC 3.15414 T	China: Tibet	leaf sample	KP765384	KP765380	KP765382	MN969164
*T. rapidus* Guevara-Suarez et al. 2017	CBS 142382 T	USA	human bronchoalveolar lavage	LT558970	LT559087	LT795600	LT795601
*T. ruber* (Stoll) N. Yilmaz et al. 2012	CBS 132704 T	UK	aircraft fuel tank	JX315662	JX315629	KF741938	JX315700
*T. rubicundus* (J.H. Mill. et al.) Samson et al. 2011	CBS 342.59 T	USA	soil	JN899384	JX494309	KF741956	KM023296
*T. sayulitensis* Visagie et al. 2014	CBS 138204 T	Mexico	house dust	KJ775713	KJ775206	KJ775422	MN969146
*T. siamensis* (Manoch and C. Ramírez) Samson et al. 2011	CBS 475.88 T	Thailand	forest soil	JN899385	JX091379	KF741960	KM023279
*T. soli* Jurjević and S.W. Peterson 2019	NRRL 62165 T	USA	soil	MH793074	MH792947	MH793011	MH793138
*T. sparsus* L. Wang 2021	CGMCC 3.16003 T	China: Beijing	soil	MT077182	MT083924	MT083925	MT083926
*T. stellenboschiensis* Visagie and K. Jacobs 2015	CBS 135665 T	South Africa	soil	JX091471	JX091605	JX140683	MN969157
*T. stipitatus* (Thom) C.R. Benj. 1955	CBS 375.48 T	USA	rotting wood	JN899348	KM111288	KF741957	KM023280
*T. stollii* N. Yilmaz et al. 2012	CBS 408.93 T	Netherlands	AIDS patient	JX315674	JX315633	JX315646	JX315712
*T. striatoconidium* (R.F. Castañeda and W. Gams) Houbraken et al. 2020	CBS 550.89 T	Cuba	leaf litter of *Pachyanthus poirettii*	MN431418	MN969441	MN969360	MT156347
*T. thailandensis* Manoch et al. 2013	CBS 133147 T	Thailand	forest soil	JX898041	JX494294	KF741940	KM023307
*T. tumuli* Jurjević and S.W. Peterson 2019	NRRL 62151 T	USA	soil from prairie	MH793071	MH792944	MH793008	MH793135
*T. veerkampii* Visagie et al. 2015	CBS 500.78 T	Columbia	soil	KF741984	KF741918	KF741961	KX961279
*T. verruculosus* (Peyronel) Samson et al. 2011	NRRL 1050 T	USA	soil	KF741994	KF741928	KF741944	KM023306
*T. versatilis* Bridge and Buddie 2013	IMI 134755 T	UK	unknown	MN431395	MN969412	MN969319	MN969161
*T. viridis* (Stolk and G.F. Orr) Arx 1987	CBS 114.72 T	Australia	soil	AF285782	JX494310	KF741935	JN121430
*T. viridulus* Samson et al. 2011	CBS 252.87 T	Australia	soil	JN899314	JX091385	KF741943	JF417422
*T. xishaensis* X.C. Wang et al. 2016	CGMCC 3.17995 T	China: Hainan	soil	KU644580	KU644581	KU644582	MZ361364
*T. trachyspermus* (Shear) Stolk and Samson 1973	CBS 373.48 T	USA	unknown	JN899354	KF114803	KJ885281	JF417432

**Table 3:** New species and newly generated sequences reported in this study.

Species	Strain	Locality	Substrate	ITS	BenA	CaM	RPB2
*T. chongqingensis* X.C. Wang and W.Y. Zhuang sp. nov.	CS26-67 T	China: Chongqing	soil	MZ358001	MZ361343	MZ361350	MZ361357
	CS26-63	China: Chongqing	soil	MZ358002	MZ361344	MZ361351	MZ361358
	CS26-73	China: Chongqing	soil	MZ358003	MZ361345	MZ361352	MZ361359
	CS26-75	China: Chongqing	soil	MZ358004	MZ361346	MZ361353	MZ361360
*T. wushanicus* X.C. Wang and W.Y. Zhuang sp. nov.	CS17-05 T	China: Chongqing	soil	MZ356356	MZ361347	MZ361354	MZ361361
	CS17-04	China: Chongqing	soil	MZ356357	MZ361348	MZ361355	MZ361362
	CS17-06	China: Chongqing	soil	MZ356358	MZ361349	MZ361356	MZ361363

**Table 4:** Detailed characteristics of the datasets.

Section	Loci	No. of Seq.	Length of Alignment	Model for BI
*Trachyspermi*	BenA	35	533	TVM+I+G
	CaM	34	656	SYM+I+G
	RPB2	34	920	GTR+I+G
*Talaromyces*	BenA	79	490	TrN+I+G
	CaM	79	565	SYM+I+G
	RPB2	78	978	TVM+I+G

Full names of the used models: GTR+I+G (General Time Reversible with Invariant sites and Gamma distribution); SYM+I+G (Symmetrical model with Invariant sites and Gamma distribution); TrN+I+G (Tamura–Nei model with Invariant sites and Gamma distribution); TVM+I+G (Transversion model with Invariant sites and Gamma distribution).

**Table 5:** Cultural and morphological comparisons of new species and their closely related species.

Species	CYA 25 °C (mm)	CYA 37 °C (mm)	MEA (mm)	YES (mm)	Conidia Shape	Conidia Wall	Conidia Size (μm)	Reference
*T. chongqingensis*	12–13	no growth	17–18	18–19	ellipsoidal to broad fusiform	smooth	2.5–3.5 × 2–2.5	This study
*T. minioluteus*	17–18	no growth	21–22	18	ellipsoidal	smooth	2.5–4 × 1.5–2.5	[24]
*T. minnesotensis*	24–26	no growth	13–15	21–24	ellipsoidal	smooth	2.5–3.5 × 2–3	[5]
*T. udagawae*	6–8	no growth	10–11	8–9	subglobose to ellipsoidal	smooth	3–4 × 2–3	[24]
*T. cnidii*	30–35	17–20	38–43	40–45	ellipsodial	smooth to finely rough	3–4 × 2–2.5	[25]
*T. siamensis*	20–22	15	32–33	27–28	ellipsoidal to fusiform	smooth to finely rough	3–4 × 2–3	[24]
*T. wushanicus*	21–24	17–19	40–44	24–28	ellipsoidal to broad fusiform	smooth to finely rough	3–4 × 2.5–3	This study

## References

[1] Mendez-Liter J.A., Nieto-Dominguez M., Fernandez de Toro B., Gonzalez Santana A., Prieto A., Asensio J.L., Canada F.J., de Eugenio L.I., Martinez M.J. (2020). A glucotolerant β-glucosidase from the fungus *Talaromyces amestolkiae* and its conversion into a glycosynthase for glycosylation of phenolic compounds. Microb. Cell Fact..

[2] Inoue H., Decker S.R., Taylor L.E., Yano S., Sawayama S. (2014). Identification and characterization of core cellulolytic enzymes from *Talaromyces cellulolyticus* (formerly *Acremonium cellulolyticus*) critical for hydrolysis of lignocellulosic biomass. Biotechnol. Biofuels.

[3] Morales-Oyervides L., Ruiz-Sanchez J.P., Oliveira J.C., Sousa-Gallagher M.J., Mendez-Zavala A., Giuffrida D., Dufosse L., Montanez J. (2020). Biotechnological approaches for the production of natural colorants by *Talaromyces*/*Penicillium*: A review. Biotechnol. Adv..

[4] Frisvad J.C., Yilmaz N., Thrane U., Rasmussen K.B., Houbraken J., Samson R.A. (2013). *Talaromyces atroroseus*, a new species efficiently producing industrially relevant red pigments. PLoS ONE.

[5] Guevara-Suarez M., Sutton D.A., Gene J., Garcia D., Wiederhold N., Guarro J., Cano-Lira J.F. (2017). Four new species of *Talaromyces* from clinical sources. Mycoses.

[6] Li L., Chen K., Dhungana N., Jang Y., Chaturvedi V., Desmond E. (2019). Characterization of clinical isolates of *Talaromyces marneffei* and related species, California, USA. Emerg. Infect. Dis..

[7] Cao C., Xi L., Chaturvedi V. (2019). Talaromycosis (Penicilliosis) due to *Talaromyces* (*Penicillium*) *marneffei*: Insights into the clinical trends of a major fungal disease 60 years after the discovery of the pathogen. Mycopathologia.

[8] Houbraken J., Kocsube S., Visagie C.M., Yilmaz N., Wang X.C., Meijer M., Kraak B., Hubka V., Bensch K., Samson R.A. (2020). Classification of *Aspergillus*, *Penicillium*, *Talaromyces* and related genera (Eurotiales): An overview of families, genera, subgenera, sections, series and species. Stud. Mycol..

[9] Doilom M., Guo J.W., Phookamsak R., Mortimer P.E., Karunarathna S.C., Dong W., Liao C.F., Yan K., Pem D., Suwannarach N. (2020). Screening of phosphate-solubilizing fungi from air and soil in Yunnan, China: Four novel species in *Aspergillus*, *Gongronella*, *Penicillium*, and *Talaromyces*. Front. Microbiol..

[10] Sun B.D., Chen A.J., Houbraken J., Frisvad J.C., Wu W.P., Wei H.L., Zhou Y.G., Jiang X.Z., Samson R.A. (2020). New section and species in *Talaromyces*. MycoKeys.

[11] Crous P.W., Cowan D.A., Maggs-Kolling G., Yilmaz N., Larsson E., Angelini C., Brandrud T.E., Dearnaley J.D.W., Dima B., Dovana F. (2020). Fungal Planet description sheets: 1112–1181. Persoonia.

[12] Wei S., Xu X., Wang L. (2021). Four new species of *Talaromyces* section *Talaromyces* discovered in China. Mycologia.

[13] Visagie C.M., Houbraken J., Frisvad J.C., Hong S.B., Klaassen C.H., Perrone G., Seifert K.A., Varga J., Yaguchi T., Samson R.A. (2014). Identification and nomenclature of the genus *Penicillium*. Stud. Mycol..

[14] Wang X.C., Chen K., Xia Y.W., Wang L., Li T.H., Zhuang W.Y. (2016). A new species of *Talaromyces* (Trichocomaceae) from the Xisha Islands, Hainan, China. Phytotaxa.

[15] Wang X.C., Chen K., Qin W.T., Zhuang W.Y. (2017). *Talaromyces heiheensis* and *T. mangshanicus*, two new species from China. Mycol. Prog..

[16] Wang X.C., Chen K., Zeng Z.Q., Zhuang W.Y. (2017). Phylogeny and morphological analyses of *Penicillium* section *Sclerotiora* (Fungi) lead to the discovery of five new species. Sci. Rep..

[17] Katoh K., Standley D.M. (2013). MAFFT multiple sequence alignment software version 7: Improvements in performance and usability. Mol. Biol. Evol..

[18] Hall T.A. (1999). BioEdit: A user-friendly biological sequence alignment editor and analysis program for Windows 95/98/NT. Nucl. Acids. Symp. Ser..

[19] Tamura K., Stecher G., Peterson D., Filipski A., Kumar S. (2013). MEGA6: Molecular evolutionary genetics analysis version 6.0. Mol. Biol. Evol..

[20] Stamatakis A. (2006). RAxML-VI-HPC: Maximum likelihood-based phylogenetic analyses with thousands of taxa and mixed models. Bioinformatics.

[21] Miller M.A., Pfeiffer W., Schwartz T. Creating the CIPRES Science Gateway for Inference of Large Phylogenetic Trees. Proceedings of the Gateway Computing Environments Workshop (GCE).

[22] Ronquist F., Teslenko M., van der Mark P., Ayres D.L., Darling A., Hohna S., Larget B., Liu L., Suchard M.A., Huelsenbeck J.P. (2012). MrBayes 3.2: Efficient Bayesian phylogenetic inference and model choice across a large model space. Syst. Biol..

[23] Posada D., Crandall K.A. (1998). MODELTEST: Testing the model of DNA substitution. Bioinformatics.

[24] Yilmaz N., Visagie C.M., Houbraken J., Frisvad J.C., Samson R.A. (2014). Polyphasic taxonomy of the genus *Talaromyces*. Stud. Mycol..

[25] Sang H., An T.J., Kim C.S., Shin G.S., Sung G.H., Yu S.H. (2013). Two novel *Talaromyces* species isolated from medicinal crops in Korea. J. Microbiol..

[26] Marchese C. (2015). Biodiversity hotspots: A shortcut for a more complicated concept. Glob. Ecol. Conserv..

[27] Zhang Y.B., Wang G.Y., Zhuang H.F., Wang L.H., Innes J.L., Ma K.P. (2021). Integrating hotspots for endemic, threatened and rare species supports the identification of priority areas for vascular plants in SW China. Forest Ecol. Manag..

